# Charting everyday activities in later life: Study protocol of the mobility, activity, and social interactions study (MOASIS)

**DOI:** 10.3389/fpsyg.2022.1011177

**Published:** 2023-01-24

**Authors:** Christina Röcke, Minxia Luo, Pia Bereuter, Marko Katana, Michelle Fillekes, Victoria Gehriger, Alexandros Sofios, Mike Martin, Robert Weibel

**Affiliations:** ^1^University Research Priority Program ‘Dynamics of Healthy Aging’, University of Zurich, Zurich, Switzerland; ^2^Center for Gerontology, University of Zurich, Zurich, Switzerland; ^3^Department of Psychology, University of Zurich, Zurich, Switzerland; ^4^Institute of Geomatics, University of Applied Sciences and Arts Northwestern Switzerland, Muttenz, Switzerland; ^5^Department of Geography, University of Zurich, Zurich, Switzerland

**Keywords:** mobility, physical activity, social interactions, cognitive activities, ambulatory assessment, functional ability, aging

## Abstract

Prominent theories of aging emphasize the importance of resource allocation processes as a means to maintain functional ability, well-being and quality of life. Little is known about which activities and what activity patterns actually characterize the daily lives of healthy older adults in key domains of functioning, including the spatial, physical, social, and cognitive domains. This study aims to gain a comprehensive understanding of daily activities of community-dwelling older adults over an extended period of time and across a diverse range of activity domains, and to examine associations between daily activities, health and well-being at the within- and between-person levels. It also aims to examine contextual correlates of the relations between daily activities, health, and well-being. At its core, this ambulatory assessment (AA) study with a sample of 150 community-dwelling older adults aged 65 to 91 years measured spatial, physical, social, and cognitive activities across 30 days using a custom-built mobile sensor (“uTrail”), including GPS, accelerometer, and audio recording. In addition, during the first 15 days, self-reports of daily activities, psychological correlates, contexts, and cognitive performance in an ambulatory working memory task were assessed 7 times per day using smartphones. Surrounding the ambulatory assessment period, participants completed an initial baseline assessment including a telephone survey, web-based questionnaires, and a laboratory-based cognitive and physical testing session. They also participated in an intermediate laboratory session in the laboratory at half-time of the 30-day ambulatory assessment period, and finally returned to the laboratory for a posttest assessment. In sum, this is the first study which combines multi-domain activity sensing and self-report ambulatory assessment methods to observe daily life activities as indicators of functional ability in healthy older adults unfolding over an extended period (i.e., 1 month). It offers a unique opportunity to describe and understand the diverse individual real-life functional ability profiles characterizing later life.

## 1. Introduction

Daily activities have been proposed to play a pivotal role in health and well-being in numerous aging theories (Martin et al., 2015; Michel and Sadana, 2017). For example, the activity theory by Havighurst (1963) proposes that a key condition for older adults to obtain satisfaction and happiness is to maintain the activities of middle age as long as possible and to find substitutes for those activities they have to give up. Rowe and Kahn (1997) emphasized that successful aging is not only about the absence of disease or the maintenance of functional physical and cognitive capacities, but also about active engagement in social and productive activities. In promoting healthy and active aging, the World Health Organization (WHO) has also placed high emphasis on daily activities. Specifically, active aging is defined as “the process of optimizing opportunities for health, participation, and security for the enhancement of quality of life” (World Health Organization, 2002, p. 12). Moreover, the recent healthy aging model (World Health Organization, 2020) emphasizes the enablement of older adults to be mobile, have relationships, and contribute to society as a reflection of functional ability and, in turn, of healthy aging at many levels of functioning.

Studies that invited older adults to report their time use during the preceding day (i.e., “yesterday interview,” day reconstruction method) have shown that a typical day of many older adults consists of (1) self-maintenance activities [measured by Activities of Daily Living (ADLs), and Instrumental Activities of Daily Living (IADLs)], (2) resting (e.g., sleep), and (3) various physical, social, cognitive, and spatial activities (Horgas et al., 1998; Möwisch et al., 2022). This third type of activities has also been referred to as leisure activities or lifestyle activities, reflecting the idea that older adults make agentic choices in their leisure time, which, eventually, form their lifestyle (Jopp and Hertzog, 2010; Bielak, 2017). Lifespan developmental theory explicitly conceptualizes individuals as producers of their own development, flexibly adapting to biological and environmental opportunities and constraints (Baltes et al., 1998). In turn, examining how older adults allocate their time and energy to different activities, particularly to lifestyle activities, helps to understand how individuals “produce” their own developmental pathways and achieve the maintenance of their individual health and well-being in older age.

To embrace the advancement of mobile technology and to comprehensively examine daily activities, this study aims to obtain a comprehensive description and understanding of daily activities of community-dwelling older adults in relation to a wide range of within-person (i.e., intraindividual) and between-person (i.e., interindividual) differences in health and well-being by combining different ambulatory assessment methods (i.e., passive activity sensing that requires no input from the person and experience-sampling that involves self-reports). More specifically, this study developed a single custom-built sensor (i.e., the “uTrail”) that includes a Global Positioning System (GPS), an accelerometer, and audio recording for high-density continuous and simultaneous measurements throughout a full day without the need to recharge. The uTrail was used to observe 150 older adults over 30 days in 2018. In the first 2 weeks, a smartphone was used in addition to the uTrail to concurrently collect a wide range of data seven times per day, including self-reported daily activities, mood, stress, and context information as well as working memory performance. The combination of different sensors and self-reports provides a rich collection of both objective and subjective momentary information on diverse activities and their contextual correlates. In sum, this is the first study which combines multi-domain activity sensing and experience-sampling to observe healthy older adults over an extended daily life period (i.e., up to 1 month), providing a unique opportunity to better understand daily activities and their relation to health and well-being as indicators of functional ability in healthy aging.

### 1.1. Capturing daily activities, health and well-being in older age through the lens of ambulatory assessment

Decades of research have shown that more active engagement in daily activities is closely associated with older adults’ health and well-being (Hertzog et al., 2008; Adams et al., 2011). In recent years, ambulatory assessment methods have become more popular in assessing daily activities in older adults in psychological research (Brose and Ebner-Priemer, 2015; Conner and Mehl, 2015). Specifically, ambulatory self-report is the method with which individuals repeatedly provide responses to queries on their current or very recent behaviors and experiences (Conner and Mehl, 2015). Ambulatory self-report has been used to examine a wide range of psychological constructs in diverse age groups, including daily activities, functional health, and well-being. For example, activities (e.g., TV watching, social, physical activities) that were reported multiple times per day over days are associated with interindividual differences in baseline self-reported pain and physical health (Fingerman et al., 2021). At the intraindividual level, self-reported activities are also associated with concurrent emotions and cognitive performance (Pauly et al., 2017; Campbell et al., 2020). The ambulatory self-report method, prompting participants to report activities momentarily or shortly beforehand, has the advantage of minimizing retrospective recall bias on daily activities (Hatt et al., 2021). It also enables examination of interindividual differences in activity engagement as well as of intraindividual variations in daily activities over time and contexts (Hamaker, 2012). Most studies with ambulatory self-report have lasted over only 1 or 2 weeks. Although this may have reduced participant burden, it was probably insufficient to capture an extended view of the typical daily routine of an older adult and their agentic role in “producing” their own life.

Passive sensing technology (a different ambulatory assessment method), sharing the advantages of ambulatory self-report in minimizing retrospective recall bias and enabling analyses on inter- and intra-individual differences, overcomes the disadvantage of self-report by objectively and unobtrusively monitoring daily activities (Harari et al., 2017; Macdonald et al., in press). Audio recordings, for instance, have been used to observe spoken conversations, reflecting social and cognitive activities in daily life (Demiray et al., 2020). Time spent in conversations was associated with inter- and intra-individual differences in well-being across adulthood (Milek et al., 2018; Sun et al., 2019). Similarly, physical activities assessed by accelerometers have been shown to be associated with well-being and cognitive abilities on the inter- (Dillon et al., 2018; Burzynska et al., 2020) and intra-individual levels (Maher and Conroy, 2017) in samples of young, middle-aged, and older adults. Using a GPS tracking kit to assess older adults’ spatial activities, studies have examined time out-of-home in relation to inter- and intraindividual differences in well-being and physical and cognitive health (Kaspar et al., 2015; Petersen et al., 2015; Kamalyan et al., 2021).

Daily activities in aging research have been quantified often, if not primarily, by frequency and type (Bielak et al., 2019). Passive sensing technology offers a chance to largely enrich dimensions of daily activities in research, which would otherwise only be accessible through detailed interviews with participants. For example, audio recordings offer rich information on the nature of daily conversations (Mehl and Pennebaker, 2003). Specifically, conversation content, relating to reminiscence or future- and past-time orientation, have been shown to differ by age between young and older adults (Demiray et al., 2018; Haas et al., 2022) and by interindividual differences in well-being (Brianza and Demiray, 2019; Demiray et al., 2019). Language features extracted from audio recordings, such as use of complex language, have also been shown to be associated with interindividual differences in chronological age (Luo et al., 2019, 2020), executive functioning (Polsinelli et al., 2020), and working memory (Ferrario et al., 2022). Indicators of physical activities could be diverse, including volume (e.g., time spent on walking, step count), patterns (e.g., length of walking bouts), and variability of bout lengths, which were shown to relate to older adults’ cognitive health (Mc Ardle et al., 2020). Similarly, mobility has also been shown to be a multidimensional concept (much more complex than “time out-of-home”), whose relations with health and well-being require much more research to investigate (Wettstein et al., 2014; Fillekes et al., 2019a).

Furthermore, passive sensing technology offers rich data streams with which activity patterns involving temporal structures could be discovered. First, studies with ambulatory self-report methods assessing activities multiple times per day have shown that moments of activity engagement are associated with not only concurrent, but also subsequent emotions, fatigue, and cognitive performance with delayed time lags (Paolillo et al., 2018; Zhaoyang et al., 2021). With more closely-spaced sampling, studies using passive sensing technology have great potential to uncover time-lagged associations between activities and psychological outcomes. Second, closely-spaced data from sensing technology also enable research to uncover novel activity patterns in time series, such as sequential complexity (Paraschiv-Ionescu et al., 2012), temporal regularity (D’Mello and Gruber, 2021), and time-lagged patterns and trajectories (Kuppens and Verduyn, 2017). Additionally, large volumes of daily activities data from sensing technology enable big data analytics, such as using machine learning methods in predicting older adults’ health and well-being (Ferrario et al., 2020, 2022).

Thus far, most studies have used one type of technology to study a specific type of activity. Some have combined ambulatory self-report and one sensing technology (Sun et al., 2020; Haas et al., 2022). Very few of them have combined different sensing technologies together with ambulatory self-report to obtain contextualized multi-domain assessments. Nevertheless, in spite of its advantages, activities captured by sensing technology could sometimes require extra works on validating and analyzing, to establish their links with subjective psychological experience (Harari et al., 2017; Cornet and Holden, 2018). Thus, combining ambulatory self-report and different passive sensing technologies to simultaneously capture different types of activities and observe how they unfold and interact with each other in daily life for a longer period can extract more comprehensive information of daily activities and their relations to within- and between-person health variation.

### 1.2. Adding a layer: Contextual correlates of daily activities

One of the salient advantages of ambulatory assessment methods over single-occasion retrospective self-report measurements is the possibility to examine contextual factors that co-occur with an activity (Shiffman et al., 2008; Brose and Ebner-Priemer, 2015). Social and physical contexts have been conceptualized as environmental opportunities or barriers for activities participation, which subsequently influence health and well-being (Stine-Morrow et al., 2014; Wahl and Gerstorf, 2018). Ambulatory assessment methods, particularly sensing technology, capture large amounts of information on contextual situations that could be examined in relation to psychological variables (Harari et al., 2020; Lazarević et al., 2020).

Thus far, only a few ambulatory assessment studies have examined effects of contextual factors on associations between daily activities and health and well-being. For example, findings from a 14-day study with GPS and accelerometer assessment in older adults showed that, at the between-person level, time spent outdoors was significantly associated with steps-per-day, which were associated with baseline cardiorespiratory fitness, lower-extremity strength, and well-being (Harada et al., 2017). The authors proposed that their findings suggest outdoor contextual environments influence physical and psychological functioning through stimulating physical activities. Fingerman et al. (2021) showed that older adults who reported more baseline pain and lower physical functioning had a higher likelihood of visiting doctors, but this association was only significant when social encounters were present at the assessment points. Thus, the context of who is present in the moment would influence associations between activities and physical health. Luo et al. (2019, 2020) showed that, within persons, older adults produced equally complex grammatical structures when talking with the significant other as younger adults, but simpler grammar than younger adults when talking with strangers. Their findings suggest that being in familiar contexts, such as with emotionally close others, enables (and possibly motivates) older adults to produce more complex language. In sum, the above findings highlight the importance of contextual correlates on daily activities in various domains, including physical, cognitive, and social activities. Further examination of contextual effects on associations between daily activities and health and well-being in older age is desired to describe and understand the diversity of daily life pathways to functional health during adulthood and aging. With both self-report and objectively measured contexts of daily activities combined, understanding of daily activities would become much more comprehensive.

### 1.3. The current study

The Mobility, Activity, and Social Interactions Study (MOASIS) is an interdisciplinary study at the University of Zurich’s (UZH) Research Priority Program (URPP) “Dynamics of Healthy Aging” that aims to understand daily activity profiles of healthy older adults and examine intra- and interindividual associations between these profiles, health, and well-being. As such, it also aims to examine contextual determinants of associations between daily activities and health and well-being in older age.

**Figure 1:**
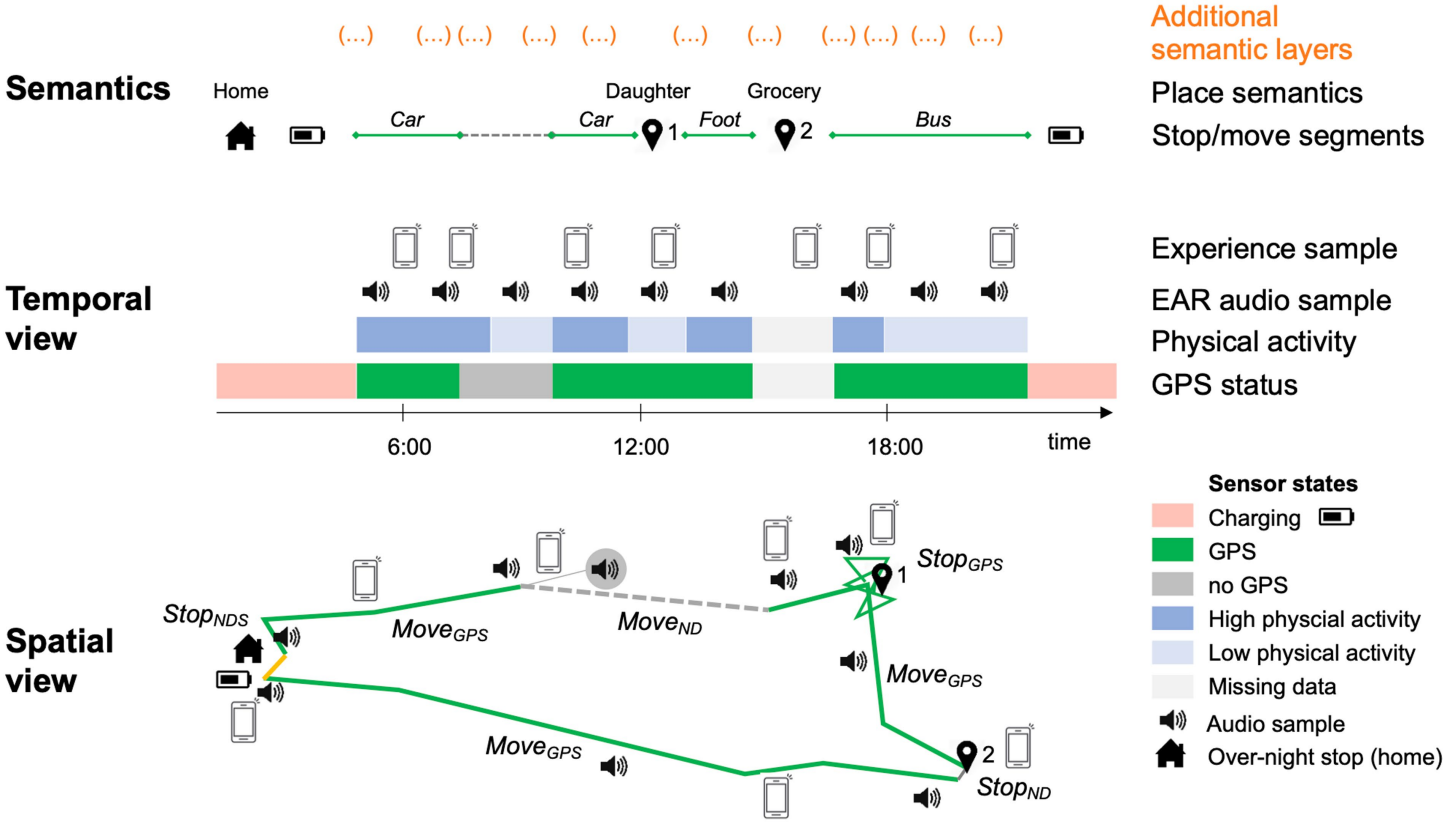
Reconstruction of spatio-temporal daily life trajectories.

We expect to observe diverse activity profiles in different healthy older adults and will examine associations between daily activities, health, and well-being. We will also examine effects of contextual factors on the associations between daily activities, health, and well-being at both the within-person and between-person levels. Furthermore, with the high-density sensor-based spatial and physical activity data, we expect to develop innovative measures and apply advanced statistical models to understand patterns of daily activities of healthy older adults. Figure 1, for instance, displays a visualization of one potential outcome of this study, in which a comprehensive representation of contextualized activities across multiple activity domains is linked over temporal and spatial contexts as one basis for the identification of the kinds of typical real-life activities of different individuals that unfold and how they can then be linked with different health and well-being parameters.

## 2. Methods and analysis

### 2.1. Recruitment and sample

Recruitment was carried out through the survey center of the URPP Dynamics of Healthy Aging using the internal participant database. Additional means of recruitment involved snowballing among registered participants as well as advertisements in local newspapers. Inclusion criteria for the study involved sufficient eyesight to operate the smartphone and uTrail, computer and internet access at home (assessed via self-report), absence of cognitive impairment as tested with a cognitive screening (Mini Mental State Examination Score ≥ 27), and being 65 years or older.

Eligible participants were contacted via phone or email and informed about the general goals and procedure of the study. Upon agreement, a package was sent to the participants containing an information brochure detailing the study background, assessment procedure and time requirements from the participants and compensation, as well as the informed consent form and a pre-stamped return envelope. Upon receipt of the informed consent, a technical research assistant called each participant to schedule all required study components and lab visits. A total of 161 participants enrolled in the study and 150 participants (aged 65–91 years) met all the inclusion criteria. Table 1 provides information on sociodemographic sample characteristics.

**Table 1:** Descriptive statistics of key sample characteristics.

Variables	Frequency (*N* = 150)	Mean (SD)
Age		73.41 (5.56)
Women	53%	
Marital status		
Married/long-term partnership	55%	
Widowed	14%	
Divorced	24%	
Single	7%	
Years of education		14.02 (3.34)
Living alone	41%	
Monthly income (CHF)		
≤4,000	38%	
4,001–8,000	48%	
≥8,001	14%	
Retired	83%	
Cognitive ability (MMSE; cutoff score for inclusion ≥27)		28.62 (1.10)
Self-reported health (SF-12; 1, poor – 5, excellent)		3.71 (0.81)

### 2.2. Procedure

**Figure 2:**
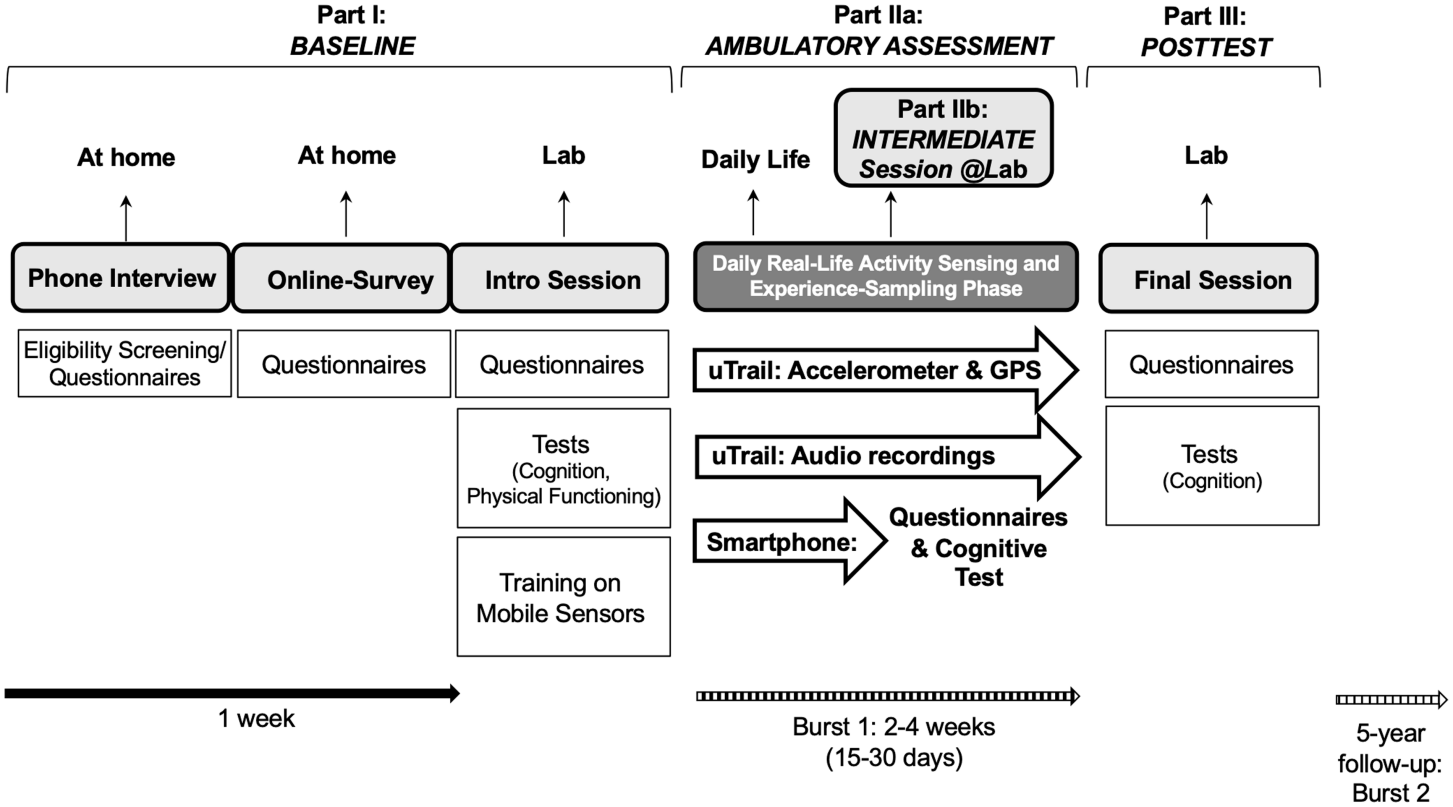
Design of the MOASIS project (first burst).

As shown in Figure 2, the MOASIS design involved (I) a 3-part baseline assessment consisting of a telephone interview, a web-based questionnaire and a lab visit, (II) a 30-day ambulatory assessment and experience sampling phase, an intermediate laboratory session 2 weeks into this ambulatory testing period used for a brief recap and mobile device return and exchange, and (III) a posttest lab session at the very end of the full 30-day ambulatory assessment phase. This core assessment design will be repeated longitudinally after 5 years.

*Part I: Baseline Assessment*. The *(a) telephone interview* consisted of questionnaires primarily assessing mobility and leisure activities as well as depressive symptoms. These questionnaires, in part, proved during pilot testing to require more detailed instructions than deemed feasible in a web-based assessment. They were assessed over the phone by trained research assistants who directly entered responses into a websurvey platform. The telephone interview was scheduled to last about 45 min. Following the telephone interview, participants received a link to *(b)* an *online survey* implemented using SoSci Survey (Leiner, 2019) to be completed at home within 1 week. This survey included a wide range of questionnaires assessing demographic information, living circumstances in the home and the neighborhood important for mobility and physical, social, and cognitive activity, as well as trait ratings of constructs representing key facets of quality of life and psychological adaptation (see Table 2). We included three single items as attention controls throughout this long questionnaire to identify participants who may have just clicked through the items (e.g., “When reading this sentence, please check 2 on the rating scale.,” “Which of the following items resembles an apple most closely?”). One week after the telephone interview, participants attended a *(c)* lab-based *introductory session* at UZH during which they completed a psychometric cognitive test battery of a wide range of crystallized and fluid cognitive abilities as well as a physical performance test. Participants were also familiarized with the uTrail and the smartphone questionnaire and cognitive testing.

**Table 2:** Questionnaire measures and laboratory tasks across assessments.

**Constructs and variables**	BL	IM	PT	Momentary/Daily	Reference
Sociodemographic variables	x				
Physical health					
**Self-rated health** (SF-12)	x		(x)	(x)	Lawton and Brody (1969); Ware et al. (1996)
Number of chronic illnesses	x				Self-developed
Vision & Hearing	x				Self-developed
Alcohol consumption	x				Self-developed
Use of walking aid	x				Self-developed
**Momentary & Daily pain**				x	Self-developed
Short Physical Performance Battery (SPPB)	x				Guralnik et al. (1994)
Sleep					
**Sleeping habits and sleeping quality**				x	Self-developed
Living Circumstances					
Living/Housing situation	x				Adapted from Iwarsson et al. (2004); Mollenkopf et al. (2004)
Attachment with own home, environment and type of living	x		x		Adapted from Iwarsson et al. (2004); Mollenkopf et al. (2004)
Neighborhood Environment Walkability Scale (NEWS)	x				Bödeker and Bucksch (2011); Saelens et al. (2003)
Typical weekly routine activities	x				Self-developed
Mobility and Context					
**(Daily) Life space** (LSQ)	x		x	x	Stalvey et al. (1999)
Favorite places	x		x		Adapted from Iwarsson et al. (2004); Mollenkopf et al. (2004)
Mobility preferences and typical mobility behavior	x		x		Self-developed
**Momentary mobility/mode of transport**				x	Self-developed
**Momentary situation, environmental & social context**				x	Self-developed
**Atypical daily locations**				x	Self-developed
Ambulatory assessment period representative of typical daily life			x		Self-developed
Physical and leisure activity					
**Physical activity** (Short IPAQ)	x		(x)	(x)	Craig et al., 2003
Leisure activities	x				Adpated from Jopp and Hertzog (2010)
**Current activity & its physical and mental effortfulness**				x	Self-developed
Subjective well-being, stress, and emotion regulation					
General Depression Scale (ADS)	x				Riediger et al. (1998)
**(Daily) Life satisfaction (SWLS)**	x		x	(x)	E. D. Diener et al. (1985)
**Positive and Negative Affect (PANAS; MDBF)**	x	x	x	x	Watson et al. (1988); Steyer et al. (1997), Grühn et al. (2010)
Psychological Well-Being	x				Ryff (1989)
**Momentary and Daily Stress (DISE)**				x	Based on Almeida et al. (2002), Wrzus et al. (2015); self-developed
**Momentary and Daily Uplifts**				x	Based on Almeida et al. (2002), Wrzus et al. (2015); self-developed
**Anticipated daily stressors and positive events**				x	Based on Scott et al. (2013)
**(Momentary) Emotion Regulation (ERQ)**	x		x	(x)	Abler and Kessler (2009); Gross and John (2003); Brans et al. (2013)
**(Daily) Psychological Need Satisfaction (BMPN)**	x			(x)	Sheldon and Hilpert (2012), Deci and Ryan (1985)
Emotional facial expression	x				Carvalho et al. (2012)
Self and personality					
Big Five Inventory (BFI-2)	x				Danner et al. (2016)
General Self-Efficacy Scale (GSE)	x		x		Schwarzer and Jerusalem (1999)
Sense of Control Scales (MIDI)	x		x		Lachman and Weaver (1998)
Mindful Attention and Awareness Scale (MAAS)	x		x		Michalak et al. (2008)
**Future Time Perspective Scale** (FTPS)	x			(x)	Allemand et al. (2012); Carstensen and Lang (1996)
Modified Balanced Time Perspective Scale (mBTPS)	x		x		Vowinckel et al. (2017)
Sense of Purpose	x				Scheier et al. (2006)
Rosenberg Self-Esteem Scale (RSE)	x				Rosenberg (1965)
Subjective age	x		x		Lindenberger et al. (2010)
Social relations					
Social contacts	x				Brunstein (1999)
Satisfaction with Social Relationships	x		x		Lindenberger et al. (2010)
Loneliness Scale (UCLA)	x				Russell et al. (1980)
Relationship Closeness Inventory (RCI)	x				Berscheid et al. (1989)
Inclusion of Other in the Self Scale (IOS)	x				Aron et al. (1992)
Berlin Social Support Scales (BSSS) and additional items	x		(x)		Butterfield and Lewis (2002); Cyranowski et al. (2013); Kasser and Ryan (1999); Schwarzer & Schulz (2003; Williams et al. (1999)
Metacognition					
Metamemory in Adulthood Questionnaire (MIA)	x		x		Dixon et al. (1988)
Cognitive Failures Questionnaire (CFQ)	x				Klumb (1995)
Thinking About Life Experience Questionnaire (TALE)	x		x		Bluck et al. (2005)
**(Momentary) Mind Wandering**	x	(x)	x	(x)	Adapted from Carriere et al. (2008) and Mrazek et al. (2011)
Daydreaming Frequency Scale	x		x		Giambra (1989)
**Momentary Temporal Focus**				x	Demiray et al. (2016)
Strategies used to solve ambulatory cognitive task		x			Self-developed
Technology					
Use of technology	x				Self-developed
Experience with technology	x				Mollenkopf et al. (2000)
Attitude towards technology	x				Mollenkopf et al. (2000)
User experience with uTrail			x		Self-developed
User experience with smartphone & ambulatory cognitive task		x			Self-developed
**(Daily) Compliance**			x	(x)	Self-developed
Cognitive ability and performance					
Verbal Knowledge	x		x		
*Vocabulary (MWT-B), spelling (LPS 1 & 2)*	x				Horn (1983), Lehrl (2005)
Verbal Fluency	x		x		
*Phonemic fluency (LPS6), semantic fluency (RWT)*					Sturm et al. (1993), Aschenbrenner et al. (2000)
Processing speed	x		x		
*Speed of perception (LPS14), digit-symbol-test (HAWIE), identical pictures test (IPS), trail making test (TMT)*					French et al. (1963), Reitan (1992), Sturm et al. (1993), von Aster and Neubauer (2009)
(Momentary) Working memory	x		x	(x)	
*Repeated numbers forward and backwards (HAWIE), 2-back task (TAP), **numeric updating task***					Zimmermann and Fimm (2002), von Aster and Neubauer (2009)
Episodic memory	x		x		
*Verbal learning test (VLMT)*					Helmstaedter et al. (2001)
Spatial memory	x		x		
*Object-Location Task*					Rasch et al. (2007)
Executive functioning	x		x		
*Switching ability in the trail making test (TMT)*					Reitan (1992)

BL = Baseline Assessment, IM = Intermediate Assessment, PT = Posttest Assessment, Daily = Daily Ambulatory Assessment Phase, (x) = variable was assessed in abbreviated version compared to Baseline (i.e., often using single items). In most instances, the acronym of the trait instrument used at BL (& PT) is provided in parentheses. Bold font highlights constructs / measures included in the ambulatory assessment phase.

**Figure 3:**
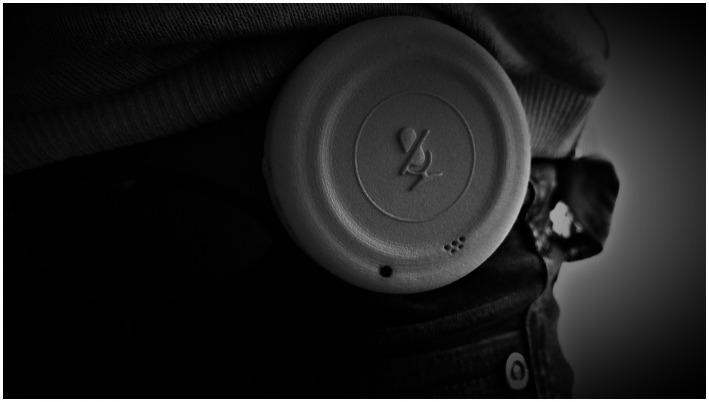
uTrail (diameter = 5.5 cm, depth = 2 cm, weight < 100 g). The uTrail has a clip to fix it on the waist (either at a belt or waistband). Photo taken by authors.

*Part IIa: Ambulatory Assessment Phase.* During this 30-day phase of daily real-life activity sensing and experience-sampling, participants wore the uTrail (Figure 3; Table 3) on their hip throughout their waking hours. They were asked to charge the device each night and there was a privacy button they could push when wanting to prevent audio recordings for the next 15 min. A red LED light indicated to participants when muting was in progress. Otherwise, participants could not and did not have to interact with the device. Activity data measured with the uTrail were stored directly on two SD cards on the device and downloaded manually at the end of Week 2 (i.e., during the intermediate session) and Week 4 (i.e., at posttest), respectively. The decision for local storage on the uTrail and manual download and against remote access and download was made for privacy reasons.

**Table 3:** uTrail mobile sensor device used in the MOASIS project.

	Construct	Sensor	Model (Manufacturer)	Variables	Sampling rate
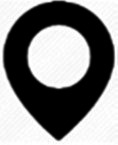	Spatial activity (i.e., mobility)	GPS	CAM-M8 (u-blox)	Timestamp, Longitude, Latitude, Satellites, Altitude, HDOP, VDOP, Speed	1/s
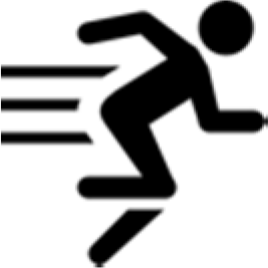	Physical activity	IMU (3-axis accelerometer, 3-axis magnetometer)	LSM303D (STMicroelectronics)	Timestamp, acceleration (x,y,z), magnetic field intensity (x,y,z)	50/s
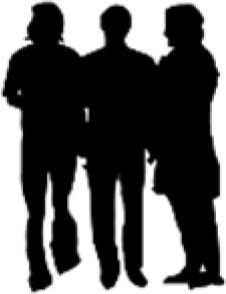	Social activity	MEMS microphone	INMP510ACEZ-R7 (InvenSense Inc.)	Timestamped 50 s – sound sample (mp3)	1/18 min

**Figure 4:**
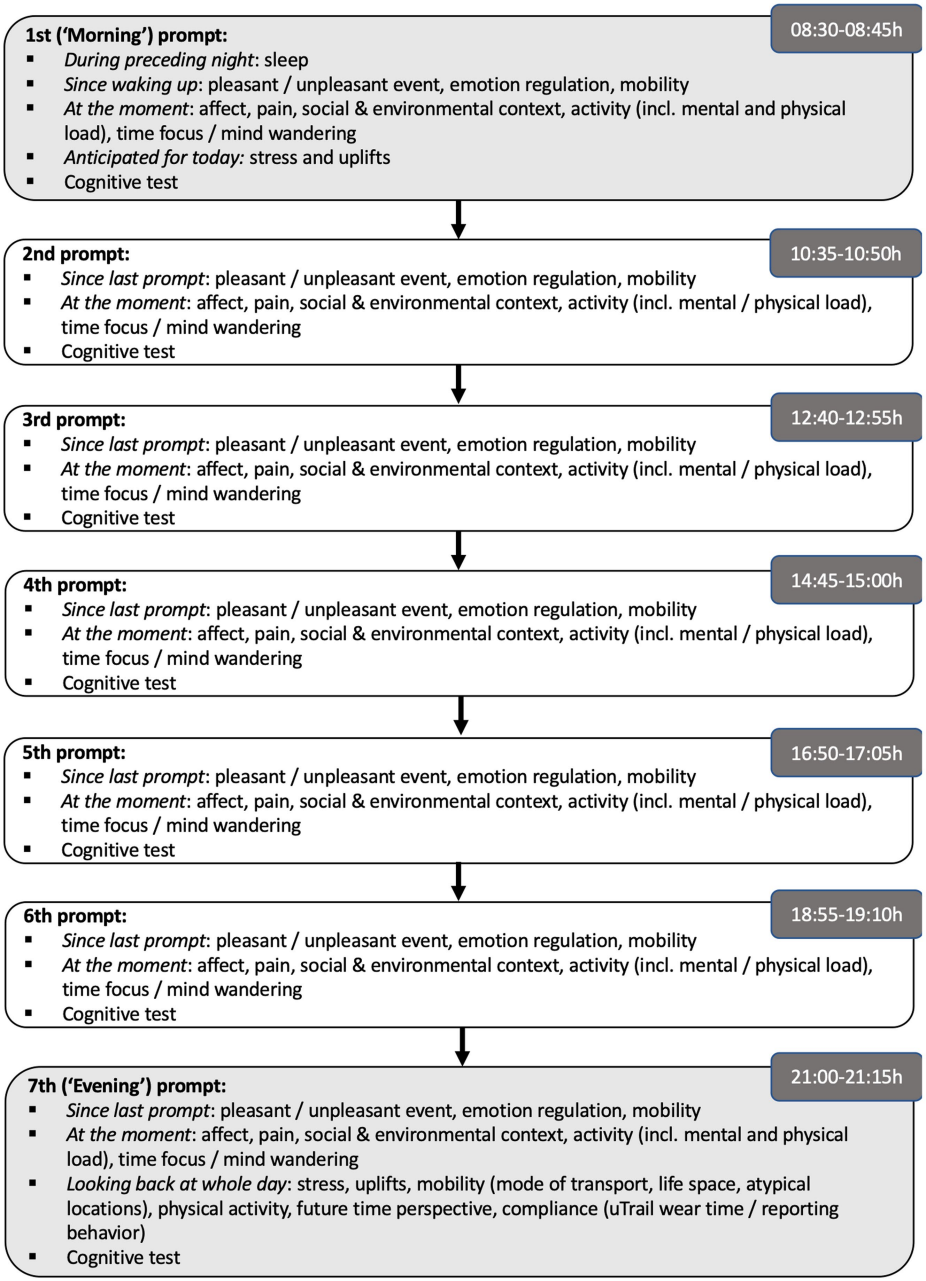
Diurnal flow of variables assessed across 7 prompts per day.

For the first 15 consecutive days of the ambulatory assessment phase (i.e., between baseline and intermediate session), participants also carried a smartphone with them and were prompted 7 times per day every 120 min with a random interval of plus or minus 0–15 min (e.g., roughly around 8:30, 10:35, 12:40, 14:45, 16:50, 18:55, and 21:00 h) to initiate a short experience-sampling and cognition test on the phone. During pilot testing, we found that this covered the waking time that our older individuals tended to report. At each prompt, participants responded to a short survey outlined in more detail below and in the end completed 2 trials of varying difficulty level of a working memory task, all of which on the smartphones provided for the study (see Figure 4 for an overview).

Participants’ compliance with the smartphone assessments was monitored online as data were transmitted to the server of the experience-sampling software used (MovisensXS). In case no experience-sampling and ambulatory cognition data collected on the smartphones were uploaded to the server, a student assistant checked in with a given participant *via* phone. Depending on the visible compliance with respect to the smartphone-based assessments, potential problems regarding both mobile devices were addressed and encouragement and appreciation for their participation was expressed.

*Part IIb: Intermediate Session*. As the uTrail can store activity data from its three sensors for up to 2 weeks, participants were invited back to the laboratory 2 weeks after the baseline lab session for data download and to return the study smartphones. Because data download and reinitialization of a uTrail device could take up to 4 h, we exchanged uTrail devices used in weeks 1–2 for a new device for each participant for the remaining second half of the ambulatory assessment period. Since the uTrails use standard commercial components that have been calibrated before deployment, inter-device variation in data accuracy is minimal, and hence device swapping is expected to have no effect on data quality. Participants also responded to a brief questionnaire on the computer regarding retrospective ratings of affect, mobility, physical activity, and mind wandering during the 2 weeks prior. Participants were also asked for a brief oral assessment of their experiences so far and then dismissed for the second half of the study.

*Part III: Posttest Session.* Thirty days after the laboratory baseline session, participants came back to the lab for a final session to return their uTrail and to complete web-based questionnaires assessing a selection of the person-level traits already measured at baseline as well as overall experience and feedback questions regarding the uTrail, the smartphone assessment, and the study in general. They also completed a slightly shortened cognitive test battery compared to the one administered at baseline. Participants could indicate whether they wanted to listen to any of their audio snippets and indicate possible audio files they wished to delete. They were then thanked and reimbursed with 150 CHF (equivalent to app. 160 $) for the entire study participation if they met compliance criteria for the ambulatory assessment phase (i.e., 80% or more of the smartphone surveys completed).

### 2.3. Measures

A battery of measures reflecting trait-like physical activities and spatial mobility, health, well-being, as well as contextual factors were collected in the baseline and posttest sessions to obtain (a) information on interindividual differences in those activity variables intensively assessed in daily life, (b) information on interindividual differences in various domains of psychological health to be investigated as antecedents, correlates, and consequences of activities carried out in daily life (emotional, cognitive, physical), and (c) information on individual environmental and other context characteristics likely to shape daily activity routines. Table 2 provides an overview of all measures and the time point or period throughout the study when they were collected.

#### 2.3.1. Baseline and posttest: Self-report measures

*Sociodemographics.* Participants provided information on conventional sociodemographic measures such as sex, age, years of education and type of occupation, marital status, presence and number of children, and income.

*Physical health.* Physical health was assessed using the Short-Form Health Survey (SF-12; Lawton and Brody, 1969; Ware et al., 1996). The survey contains questions about emotional and physical health and was slightly extended for use in the current study to include additional questions about possible head injury during the preceding 2 years, number of illnesses using a list of 23 possible diagnoses (e.g., cancer, hypertension), changes in vision and hearing as well as limitations in activities of daily living. In addition, we collected information on body mass index, consumption of alcohol, and usage of any walking aid.

*Living circumstances and mobility.* Participants indicated their living situation in more general terms (e.g., living in an urban or rural area, in an apartment or house, on which floor they live) and also regarding more specific aspects that we deemed important for our focus on physical, spatial, and social activities (e.g., whether they own a car, how many neighbors they are in contact with regularly, which means of transport they typically use). Participants reported on their typical radius of mobility using the Life Space Questionnaire (LSQ; Stalvey et al., 1999), and also indicated their five favorite locations, what they typically do at these locations and how often they visit them (self-developed based on Iwarsson et al., 2004; Mollenkopf et al., 2004) in addition to mobility preferences. We used the Neighborhood Environment Walkablity Scale (NEWS; Saelens et al., 2003; Bödeker and Bucksch, 2011) to measure individuals’ neighborhood perception (e.g., types of residences, stores and facilities, access to services, streets, places for walking and cycling, surroundings, safety from traffic, safety from crime, satisfaction with neighborhood).

*Physical and leisure activities.* We assessed typical physical activity using the short form of the International Physical Activity Questionnaire (IPAQ; Craig et al., 2003). To account for the fact that some activities, such as gardening, hiking or skiing, are more typical in some seasons than in others, these questions were asked for the warm season and the cold season separately. To obtain a broader view on individuals’ common leisure activities beyond the physical domain, participants were also asked about their typical weekly activities and routines across a number of categories (i.e., crafting, games, cultural activities, social activities, TV activities, health care and acquisition of knowledge) using an adapted version of the Leisure Activity Questionnaire by Jopp and Hertzog (2010). Participants indicated how often and at which type of location they engaged in each of these activities over the past 12 months.

*Subjective well-being, stress, and emotion regulation.* We assessed several components of subjective well-being or lack thereof, including depressive symptoms (using the German Version of the CES-D scale, the General Depression Scale [ADS]; Riediger et al., 1998), life satisfaction (Satisfaction with Life Scale [SWLS]; E. D. Diener et al., 1985), and affect. We used a large pool of items to assess high and low arousal positive and negative affect, based on the Positive and Negative Affect Schedule (PANAS; Watson et al., 1988: *upset, proud, hostile, irritable, expectant, afraid, attentive, active, strong, distressed, inspired, nervous, ashamed, guilty, interested, enthusiastic, scared, determined, alert*), the Multidimensional Affect Questionnaire (MDBF; Steyer et al., 1997: *content, rested, restless, bad, worn-out, composed, tired, great, uneasy, energetic, uncomfortable, relaxed, highly activated*), and 7 additional items to match the full range of the daily life affect experience sampling items (*without energy, balanced, sad, annoyed, angry, happy, concerned*). Psychological well-being was assessed using the Ryff-Scales (Ryff, 1989). Participants’ trait emotion regulation was measured using the Emotion Regulation Questionnaire (ERQ; Gross and John, 2003; Abler and Kessler, 2009). We further measured psychological need satisfaction (Balanced Measure of Psychological Needs Scale; Sheldon and Hilpert, 2012), and obtained behavioral measures of emotional (facial) expressions to emotional film clips using software and film clip material from Carvalho et al. (2012).

*Self and personality.* We assessed several interindividual difference characteristics from the self and personality domains, including Big Five personality traits (Big Five Inventory-2; Danner et al., 2016), self-efficacy (General Self-Efficacy Scale; Schwarzer and Jerusalem, 1999), control beliefs (Midlife Development Inventory; Lachman and Weaver, 1998), and mindfulness (Mindful Attention and Awareness Scale; Michalak et al., 2008). Furthermore, we assessed participants’ time perspective using the Future Time Perspective Scale (Carstensen and Lang, 1996) and the Modified Balanced Time Perspective Scale (mBTPS; Vowinckel et al., 2017). In addition, participants rated their sense of purpose in life (German Purpose Scale; Scheier et al., 2006), self-esteem (Rosenberg Self-Esteem Scale; Rosenberg, 1965; Von Collani and Herzberg, 2003), and several aspects of subjective age (i.e., felt age, looked age, wanted age).

*Social relations*. We assessed participants’ contacts with family members and non-family members (Brunstein, 1999), satisfaction with family and friend relationships (Lindenberger et al., 2010), as well as perceived loneliness (UCLA Loneliness Scale; Russell et al., 1980). Relationship closeness was assessed with the Relationship Closeness Inventory (RCI; Berscheid et al., 1989), and with the Inclusion of Other in Self (IOS) Scale (Aron et al., 1992). Various aspects of social support (e.g., perceived social support, support in autonomy, received support, provided support, companionship, social distress, and health related social control) were assessed using the Berlin Social Support Scales (BSSS; Schwarzer and Schulz, 2003), and adaptations from other measures (Kasser and Ryan, 1999; Williams et al., 1999; Butterfield and Lewis, 2002; Cyranowski et al., 2013).

*Metacognition.* Participants’ metacognition was measured using the Metamemory in Adulthood Questionnaire (MIA; Dixon et al., 1988), the Cognitive Failures Questionnaire (Klumb, 1995), as well as the Thinking about Life Experience (TALE) Questionnaire (Bluck et al., 2005). Mind wandering was measured using two different scales (Carriere et al., 2008; Mrazek et al., 2011), complemented by the Daydreaming Frequency Scale (Giambra, 1989).

*Technology use, experience and attitude*. Given the use of mobile technological devices for activity tracking and experience sampling, we also assessed several aspects regarding technology. On a list of 12 items (e.g., TV, phone, computer, e-reader, smartphone), participants indicated which of these technological devices they owned and how often they used these, in particular computers, smartphones, and in addition the internet. Participants also answered more specific questions about their experience with, and attitude towards technology with a set of items adapted from Mollenkopf et al. (2000). Only at posttest, we further obtained feedback on participants’ experiences and perception of the technical quality and the handling of the uTrail sensor used in this study with a set of self-developed items. Examples of items are “During the past 4 weeks to what extent have you been aware of the device in your everyday life?,” “During the past 4 weeks to what extent was the use of the device easy, intuitive?” or “During the past 4 weeks to what extent did the device influence the behavior of the people in your environment?.” Participants also responded to questions about the extent to which the uTrail and the smartphone influenced their awareness of everyday life and their environment, and the extent to which they had carried the uTrail on themselves in daily life and reasons for why not.

#### 2.3.2. Baseline and posttest: Cognitive ability and physical functioning tests

Cognitive abilities were assessed with lab-based performance tasks covering several crystallized and fluid intelligence markers. These included verbal knowledge [Mehrfachwahl-Wortschatz-Intelligenztest (MWT-B); Lehrl, 2005; spelling test from the Leistungsprüfsystem (LPS 1 & 2), Sturm et al., 1993], phonemic fluency [Leistungsprüfsystem (LPS6); Sturm et al., 1993], and semantic fluency [Regenburger Wortflüssigkeitstest (RWT); Aschenbrenner et al., 2000]. Further, we obtained interindividual differences in processing speed using four different tests [speed of perception test from Leistungsprüfsystem (LPS14); Sturm et al., 1993; digit-symbol-test from the HAWIE; von Aster and Neubauer, 2009; identical pictures test (IPS); French et al., 1963; and the trail making test (TMT), Reitan, 1992]. Working memory was assessed by the repeated numbers task forward and backwards (HAWIE; von Aster and Neubauer, 2009), a computerized 2-back task using the Test of Attentional Performance (TAP) software (Zimmermann and Fimm, 2002), and a numerical memory updating task executed on the study smartphones (Riediger et al., 2011) that is described in greater detail in the section on the ambulatory assessment of cognitive performance. Episodic memory was measured by the Verbal Learning and Memory Test (VLMT; Helmstaedter et al., 2001, a German version of Rey Auditory Verbal Learning Test (RAVLT) Rey, 1964). Spatial memory was measured by a computerized object-location task similar to the card game “Memory” (Rasch et al., 2007). Executive functioning was measured by using the difference score between version A and B of the TMT (Reitan, 1992). In addition to cognitive performance, we also assessed physical functioning with the Short Physical Performance Battery (SPPB; Guralnik et al., 1994), including performance tests for balance, walking speed, and leg strength, to obtain a baseline interindividual difference measure in these domains given our interest in capturing daily life mobility and physical activity.

#### 2.3.3. Ambulatory assessment: Passive real-life activity sensing

The uTrail used in this study for the passive sensing of spatial, physical, and social activities is a custom-built tracking device. It was developed with the aim to (a) collect multi-domain activity data in a single device, (b) provide data density in each of the three sensor units appropriate for in-depth analysis in each activity domain, (c) be able to store all that data over a period of 2 weeks, (d) provide sufficient battery capacity for at least one full day of high-density sampling, and (e) be user friendly also for an older adult population with diverse technological experiences.

As outlined in Table 3, the uTrail features the following three sensor units: a GNSS (Global Navigation Satellite System) unit (colloquially called GPS); an IMU (Inertial Measurement Unit) consisting of an accelerometer and a magnetometer; and a MEMS (microelectro-mechanical systems) microphone. All sensor units are commercial off-the-shelf components. The sampling rates were selected to reflect those commonly used for the different sensors to derive as meaningful and accurate information as possible in each activity domain. The timestamp that is recorded in all three datasets allows to cross-link information from the different sensor units. Besides timestamp, the GPS data features the position (longitude, latitude), different accuracy values (i.e., number of available satellites, horizontal and vertical dilution of precision [VDOP, HDOP, respectively]), and Doppler-based instant speed. The uTrail device stores data internally in a binary format to make best use of the available storage capacity. When the data is downloaded it is extracted and saved automatically as a Comma Separated Values (csv) text file.

The GPS recordings can be used to obtain information about an individual’s mobility patterns and multiplace personal exposures (Dodge et al., 2009; Siła-Nowicka et al., 2016; Chaix, 2018). Over 20 corresponding indicators of daily spatial mobility have been developed and coded in the R statistics system in a preceding project using data recorded with smartphones but with the same sampling and accuracy specifications as in MOASIS (Fillekes et al., 2019b).

The IMU measures acceleration and the magnetic field intensity along three dimensions to capture information on physical activity intensity and activity types. In a related project, indicators of physical activity including physical activity intensity and levels, the major posture and transport-related motion activity types (sitting, standing, lying, walking, non-level walking, running and cycling), as well as step counts (Pham et al., 2018) have been developed and implemented in R, as documented in Allahbakhshi et al. (2020).

The audio data assessments were modeled after the Electronically Activated Recorder (EAR; Mehl et al., 2001) and consist of 50-s ambient sound snippets sampled every 18 min that contain human speech recordings of the participant as a sensor-based indicator of social interactions in daily life. Such data can then be analyzed regarding social behavior, using coding or transcription and automatic language processing approaches (e.g., Yordanova et al., 2019; Ferrario et al., 2020, 2022). Based on linguistic characteristics of the speech utterances, inferences on cognitive activity are also possible (e.g., Luo et al., 2019, 2020). As ground truth data for speaker identification, we obtained noise-free speech samples from each participant during the posttest session by asking participants to read a prepared brief statement out aloud which we recorded.

#### 2.3.4. Ambulatory assessment: Experience sampling

The experience sampling was carried out on study smartphones (Motorola moto G4, Android 6.0) provided to participants and aimed to capture self-report information to complement the passive sensing carried out with the uTrail. The seven momentary surveys presented to participants each day during the experience sampling phase each contained a set of core items that were identical across prompts. In addition, some questions were only relevant in the morning (e.g., sleep quality during the preceding night, outlook on the day) or evening (e.g., review of daily stress experienced that day, subjective health). In the following, we provide an overview of the experience sampling variables available, and unless particularly mentioned, these were collected seven times per day. Figure 4 provides an overview of the diurnal flow of questions.

*Sleep*. In the first momentary survey in the morning, participants rated their daily sleep quality, the time they went to bed the night before and got up that morning, and how many hours they had slept, responding to single items each.

*Situational evaluation*. Situations can be evaluated along several dimensions to assess their psychological meaning (Rauthmann and Sherman, 2016). In an effort to include such contextual information into the real-life assessments, in addition to the related information collected using the GPS, IMU and audio data, each momentary survey included measurement of stress and uplifts, positive and negative affect, the geographical and social context, as well as the activity pursued including its intellectual and physical load. In the following paragraphs, the specific variables and their measurement are described in more detail.

*Stress and uplifts*. In the morning survey, participants were asked about anticipated stress and anticipated positive events for that same day (Scott et al., 2013). At each prompt, participants also indicated whether, since they got up in the morning or since the last prompt, they experienced something unpleasant or something pleasant (Almeida et al., 2002). For each positive response, they also indicated when during the roughly past two-hour time window this event took place or whether it was still ongoing (Wrzus et al., 2015).

A more detailed assessment of daily stress occurrence and severity was obtained at each daily evening prompt with an adapted 7-item version of the Daily Inventory of Stressful Events, including an extra item on health-related stressors (DISE; Almeida et al., 2002). Similar to the stressors, daily uplifts were assessed with five items regarding both occurrence and intensity, that directly mimicked the DISE questions, focusing on uplifts instead.

*Subjective well-being*. Emotional well-being in terms of momentary positive and negative affect intensity was measured at each of the seven momentary prompts using a select set of items also included in the baseline assessment, that were drawn from the PANAS-X (Watson et al., 1988; Grühn et al., 2010) and the momentary version of the Multidimensional Mood Questionnaire (Wilhelm and Schoebi, 2007) to cover both low and high arousal items from the emotional circumplex and those that have been shown to be sensitive to momentary fluctuations (Brose et al., 2020; *content, unwell, restless, awake, without energy, nervous, relaxed, worried, happy, angry, sad, balanced, furious*). In the evening, an additional single item measured daily life satisfaction, and personal need satisfaction was assessed with three items based on the balanced measure of psychological needs (BMPN; Sheldon and Hilpert, 2012) and the General Causality Orientation Scale (Deci and Ryan, 1985). The items asked about the connection to other people, feeling competent and whether one’s own actions were based on one’s own interests and values.

*Pain and subjective health*. Occurrence of pain was assessed both at each momentary assessment and reflecting back on the day each evening with single items. In addition, a single item asking about daily subjective health was included in the evening survey.

*Emotion regulation*. Emotion regulation was measured at every prompt by asking participants to report to which extent they had applied each of six emotion regulation strategies since waking up or since the last momentary assessment (Brans et al., 2013).

*Social and environmental context*. At each prompt, participants specified whether they were with another person or not. If others were present, the type of relation was assessed (e.g., partner, family, stranger). Participants were also asked in each momentary survey, which type of location they were in (e.g., at home, indoors, outdoors, at a private or a public place), and how often they visited that place.

*Mobility and physical activity*. Being mobile (vs. not) and type of transport mode was measured by asking participants at each prompt whether they were currently in transit or not and whether they had been moving since getting up or since the last prompt. Given a positive response, they further indicated the mode of passive or active transportation used (e.g., walking, riding a bike, taking a bus or car). In the evening, participants were asked how much time they had spent on high and moderate physical activities that day and the duration of those types of activities, using a shortened version of the IPAQ (Booth et al., 1996). In addition, we asked for ratings of the duration of active and passive locomotion, and whether individuals had been to atypical locations that day (i.e., those visited less than twice a month). They also specified their daily life space by indicating how far away from their home they had been during that day (Stalvey et al., 1999).

*General activity*. At each prompt participants indicated their current activity from a list of 13 options (e.g., social interaction, resting, doctor’s appointment, reading, or other) and how long they had been engaged in it (Wrzus et al., 2015). They also rated how physically and mentally exhausting the selected activity was.

*Metacognition*. Time focus was measured with a single item on the past, present or future focus of participants’ momentary thoughts (Demiray et al., 2016). To measure momentary mind wandering, participants were asked whether their thoughts were unrelated to what they were doing at the moment of the prompt (Carriere et al., 2013). In the evening assessment, future time perspective was measured with two items addressing the perception of time and the perception of possibilities as limited or infinite (Lang and Carstensen, 2002; Allemand et al., 2012).

*Self-reported Compliance*. In the evenings, we asked individuals whether they had been wearing the uTrail continuously during the day and for possible reasons for non-wearing time (e.g., activity in water, forgetfulness). To measure compliance regarding the questionnaires, participants indicated the extent to which they had just clicked through any of the questions during that day.

#### 2.3.5. Ambulatory assessment: Cognitive test

As part of each momentary prompt, and after responding to all experience sampling questions, participants completed two trials of a numeric memory-updating task on their smartphone that has been shown to reliably assess numerical working memory performance in an ambulatory setting, including older adults (e.g., Riediger et al., 2011). The task involved a 2 × 2 grid with a total of four digits between 0 and 9. After starting the task *via* button press, the initial digits were randomly updated by an addition or subtraction between −8 and +8, which appeared consecutively in a random order in each of the four cells. Two consecutive updating operations did not appear in the same cell. Participants had to continuously update and remember the new resulting digit in each cell. After the final operation, participants had to fill in the final result in each of the four cells, correct their results if necessary, and then finish the task again *via* button press. They then received feedback on the number of correct cells in each trail (e.g., 3 out of 4 correct cells). We used the proportion correct across both trials as the momentary working memory indicator, with a possible range of values between 0 and 1, where higher scores indicated a greater number of correct answers and thus higher working memory performance. Daily working memory was calculated as the average of the momentary working memory scores across a day.

The momentary assessment at each prompt consisted of two trials: an easier task version based on Riediger et al. (2011) and a more difficult version based on Oberauer and Kliegl (2001) and Schmiedek et al. (2010). In the easier (vs. more difficult) version, presentation time was initially 6,000 ms (vs. 4,000 ms), and 3,500 ms (vs. 1,250 ms) at each of the following operations, the time between operations was 500 ms (vs. 250 ms), and there were 5 (vs. 8) operations in total. After the final operation, participants had to fill in the final result in each of the four cells by pressing the finish button, they then received feedback on the proportion of correct cells (e.g., 3 out of 4 correct cells).

#### 2.3.6. Intermediate assessment

The goal of the intermediate assessment was to use the opportunity of participants returning after the first 2 weeks of the ambulatory assessment period to swap and drop off mobile sensor devices to ask a few questions retrospectively over those first 2 weeks to check for possible immediate changes in select constructs following the intensive experience-sampling period.

*Subjective well-being*. Participants rated the frequency of experiencing each of a number of positive and negative affects with regard to the preceding 2 weeks (rather than during the past year) that were identical to the ones used at baseline and posttest.

*Mind wandering*. To assess mind wandering and its temporal focus and emotional tone during the preceding 2 weeks, participants responded to a set of 11 items selected from the larger questionnaires used at baseline.

*Smartphone handling*. Participants answered questions about their experiences using the smartphone and with the cognition task (e.g., perceived difficulty of the task and user friendliness of the ESM application). They were also asked about the use of particular strategies to solve the cognition task (e.g., ignoring certain boxes, repeating the digits to oneself or making notes). Participants also indicated whether they considered the seven prompts per day to appropriately capture their daily experiences.

*Working memory.* Participants completed 2 trials of the working memory task on the study smartphones.

*General feedback.* Additionally, participants provided oral feedback about the past 2 weeks to the research staff, which was then written down in an open format.

### 2.4. Analytic approach and power analysis

We plan to primarily use mixed-effect models to examine between- and within-person associations between activity engagement, cognitive abilities/health/well-being, and contextual correlates. Mixed-effect models flexibly and simultaneously estimate associations at the levels of between-person and within-person, as well as across-level interactions, taking into account autocorrelated within-person errors of the repeatedly assessed variables (Bolger and Laurenceau, 2013). In order to determine the sample size of the main study, we used data from one of the pilot studies of 27 older adults completing daily mobile surveys over 30 days on a select set of similar questionnaires. Specifically, we conducted Monte Carlo simulations using the “simr” package in R based on these prior pilot data, referring to existing guidance (Bolger and Laurenceau, 2013; Green and MacLeod, 2016). More specifically, we extracted means and variances of example variables by estimating the pilot data through mixed-effects models in the “lme4” package in R (Bates et al., 2007). We then calculated standardized effect sizes (i.e., Cohen’s *d*) and variances (based on variance partitioning coefficients [VPCs]) from the mixed-effects models according to the guidance of Westfall et al. (2014). Finally, based on the extracted information, we applied Monte Carlo simulations (i.e., 1,000 times) to calculate the sample size of the main study.

For example, we estimated within-person association between the repeatedly assessed variable of life satisfaction (7-point scale: 0 = not at all to 6 = very much) and occurrence of positive events (0 = no, 1 = yes); and a moderator analysis with a baseline variable of loneliness (7-point scale: 0 = does not apply at all to 6 = applies very well). The repeatedly assessed variables of life satisfaction and occurrence of positive events had about 0.4 intraclass correlation coefficients (i.e., ICCs). The ICC scores suggest a medium-to-large degree of similarity among the repeated measurements within persons, which are similar to existing studies that have repeated assessments (Arend and Schäfer, 2019). We aimed to achieve power of 0.80 with an alpha 0.05 to detect small-to-medium size of fixed effects (Cohen’s *d* = 0.2 to 0.5; Westfall et al., 2014). Thus, we arrived at the seven prompts per day over 15 days in 150 older adults for the ambulatory assessment phase. We chose to extend the activity sensing by uTrail, involving less participant burden, for an additional 2 weeks to finally cover a full 4-week cycle in order to obtain information on possible week-to-week variation in some mobility and activity patterns.

## 3. Discussion and outlook

**Figure 5:**
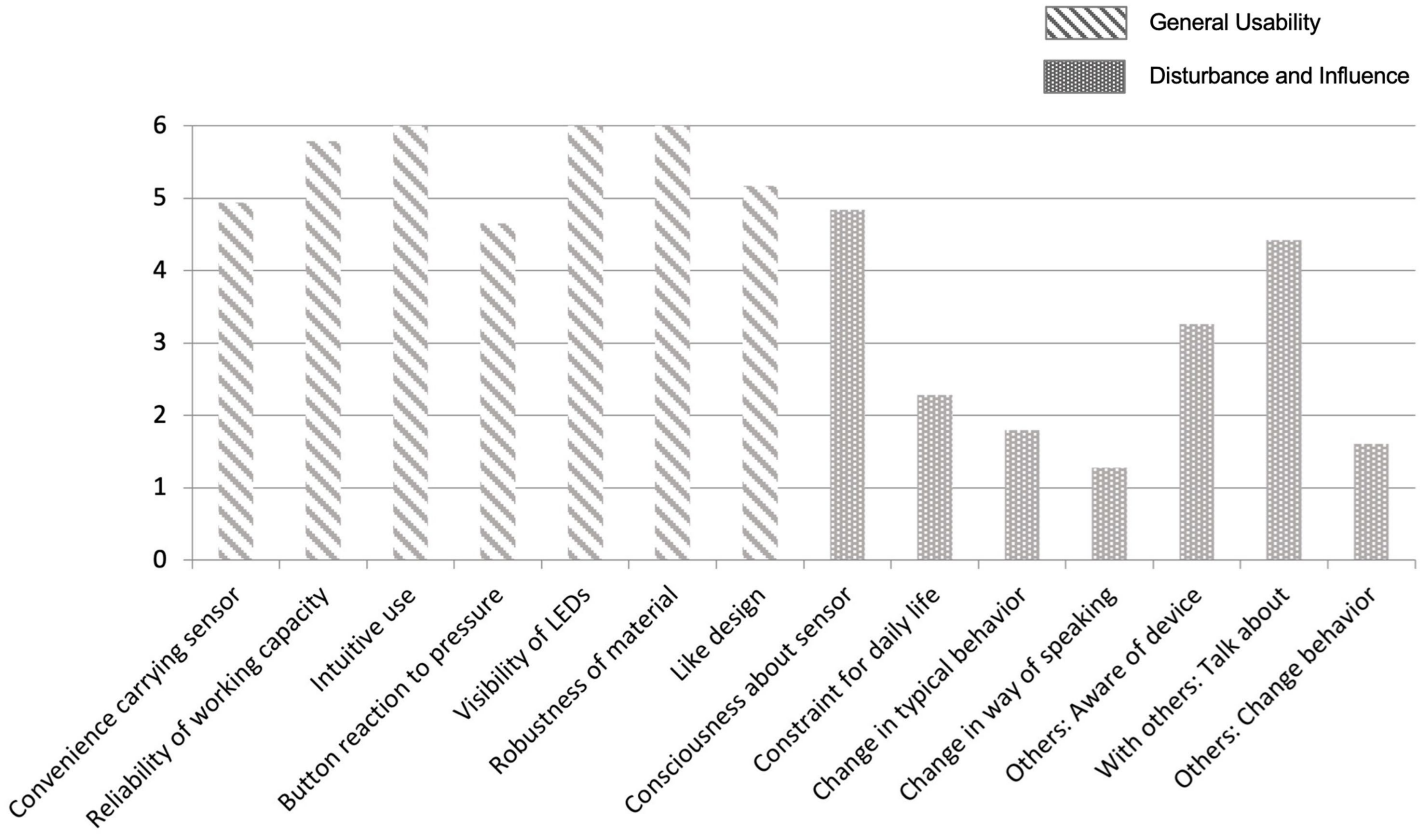
Evaluation of uTrail handling and experience by participants. Response scale ranged from 0 (does not apply at all) to 6 (applies very well).

This is the first study which combines different ambulatory assessment methods and tools (ranging from passive activity sensing to self-reports and ambulatory cognition tests) to observe community-dwelling older adults’ spatial, physical, and social activities jointly and together with well-being, health, cognition, and contextual information over up to 1 month. We have obtained a high degree of compliance in the intensive experience-sampling phase that covered a period of 2 weeks. We obtained an average of 96.67 (SD = 13.89) of the possible 105 (i.e., 7 × 15 days) prompts. Responding to the feedback questions regarding usability and experiences with the uTrail, our sample reported, on average, moderate to high usability and very low levels of disturbance and influence on daily life routines (see Figure 5). In addition, on a range of 0 (“not typical”) to 4 (“very typical), the ambulatory assessment period was rated on average as “rather typical” for participants’ life in general (M = 3.03, SD = 0.88).

This study offers unique opportunities for research into how healthy aging manifests itself in daily life activities across multiple key domains (i.e., mobility, physical activity, social interactions, cognitive activities). Sampling activity across multiple domains (and not just on physical activity, or spatial mobility) provides the added benefit of capturing a more complete picture of the ingredients of what characterizes inter- and intraindividual differences in behavioral and health-related patterns and profiles at different stages of the lifespan. It offers a unique opportunity to collect intensive objective evidence to understand daily activities across multiple domains that characterize healthy aging at the inter- and intra-individual levels. The inclusion of both passive and active sensing and self-report as well as performance data further provides a unique opportunity to establish associations between behavioral data in relation to subjective experience (Müller et al., 2020) and, potentially, to compare daily activities captured by passive sensing vs. self-report (i.e., experience sampling; Shiroma et al., 2015; Sun et al., 2019; Fillekes et al., 2019a).

Further, the 30-day long observation period of different sensing technologies offers unique opportunities to extract innovative indicators for different types of activities (Fillekes et al., 2019b; Demiray et al., 2020). The study design also enables analyses on complex time series data which is only possible with sufficient amount of data (Paraschiv-Ionescu et al., 2012; D’Mello and Gruber, 2021). These data provide a rich basis to apply a wide range of emerging analytical tools from longitudinal data analyses that address the various levels of analysis, to emerging approaches of multimodal data integration and machine learning. In particular, compared to prior studies that included only a handful of days of audio recordings (Yordanova et al., 2019; Ferrario et al., 2020, 2022), our study offers sufficient amount of data that is necessary to develop machine learning approaches that can help to automate key tasks in speech data analytics (i.e., speaker identification) for naturally occurring speech in daily real-life contexts (i.e., noisy data) and would, in turn, reduce time and personnel efforts required to (pre-)process the data.

Eventually, the first 30-day measurement period (as well as the accompanying baseline, intermediate and posttest assessments) will be repeated longitudinally to examine the interplay between daily lifestyle activity variability and long-term development. This follow-up (i.e., second burst assessment; Nesselroade, 1991), is planned for spring to summer 2023 and will include a re-assessment of the original MOASIS cohort, but in addition we aim to expand the sample by a new group of older adults with a greater range of mobility-related functional health and more diverse physical health profiles, and a young adult comparison group to examine age-related differences in activity antecedents, correlates, and outcomes over a larger portion of the adult lifespan.

With rich information of activities, health, and well-being in the current and the following bursts, our data can be used for global comparisons of healthy aging during The Decade of Healthy Aging (2021–2030) as long as the data are based on relatively unobtrusive measurement approaches. As a first effort of examining country- and thus context-specificity and comparability, we have begun to build a global network for a multi-country study on functional ability, in which region-specific modifications of the core MOASIS design are used in data collections in Hong Kong and Mexico City (World Health Organization, 2020). In sum, the MOASIS project offers data to facilitate innovative research to understand daily activities as a resource for older adults’ health and well-being.

## 4. Ethics and dissemination

The MOASIS project was conducted in compliance with the ethics regulations outlined in the Declaration of Helsinki. Participants received detailed information on the entire study procedure and all technical details in particular and signed an informed consent form in the recruitment process when agreeing to participate in the study. This study protocol was evaluated and approved by the Ethics Committee of the Faculty of Arts and Social Sciences at the University of Zurich (permission no. 17.2.4). Particular emphasis was put on ensuring privacy and data protection given the protocol involves the microlongitudinal and thus high-density collection of data from multiple sensors and sources for any given individual. All data are stored using numeric identifiers for anonymization on password-protected servers accessible only to authorized members of the research team.

Privacy protection particularly regarding the audio assessments were ensured through the following ways: (a) In the evening, the request to participants was to charge the uTrail, with the additional and explicitly outlined benefit of the uTrails being technically prevented from recording any data during the charging process. (b) In order to protect privacy during the day, participants had the possibility to activate a mute-button on the top of the device which stopped the audio sensor from recording for the following 15 min. A red LED light indicated to participants that muting was in progress. (c) The selected sampling rate ensured that only 2.5% of participants’ waking time, on average, was recorded with an average of three recordings (i.e., 2.5 min) per hour. (d) When returning the uTrail at the intermediate and posttest sessions, we asked whether prior to storage of the audio data on our servers, participants wished to screen individual audio files from a particular day and time in case of an incident or conversation they wanted to delete or at least check on. In that case, participants could listen to as many files as they wanted and then provided written consent for each of the two 2-week tracking periods, that we were allowed to download and store the audio and the other data on our storage using the numeric participant code as sole identifier. (e) We asked participants to proactively inform their social interaction partners about participation in the study and about the repeated audio recordings. We emphasized that our analytic focus was solely on the participant’s utterings, and that any transcripts and ratings focused exclusively on these and did not involve any speech utterances by non-participants.

Finally, dissemination of the study’s findings is planned within the scientific community and stakeholders from the general public and the policy sector (e.g., at the national and global level, such as Swiss Academy of Sciences, WHO).

## Ethics statement

The studies involving human participants were reviewed and approved by Ethics Committee of the Faculty of Arts and Social Sciences at the University of Zurich. The patients/participants provided their written informed consent to participate in this study.

## Author contributions

CR, RW, and MM are the principal investigators of the MOASIS study and acquired the funding. Together with PB, MK, and MF they conceived and designed the study. CR has the project management lead. Together with ML, MK, AS, and VG, CR has been coordinating the data collection campaigns. RW supervises and AS implements the uTrail development and quality assurance. CR and ML wrote the original draft of the manuscript. All authors contributed to the article and approved the submitted version.

## Funding

The research reported in this paper was supported by the Velux Stiftung (project no. 917) and the University Research Priority Program (URPP) “Dynamics of Healthy Aging” at the University of Zurich.

## Conflict of interest

The authors declare that the research was conducted in the absence of any commercial or financial relationships that could be construed as a potential conflict of interest.

## Publisher’s note

All claims expressed in this article are solely those of the authors and do not necessarily represent those of their affiliated organizations, or those of the publisher, the editors and the reviewers. Any product that may be evaluated in this article, or claim that may be made by its manufacturer, is not guaranteed or endorsed by the publisher.
